# Long Non-coding RNA XIST Attenuates Diabetic Peripheral Neuropathy by Inducing Autophagy Through MicroRNA-30d-5p/*s*irtuin1 Axis

**DOI:** 10.3389/fmolb.2021.655157

**Published:** 2021-04-28

**Authors:** Bei-Yan Liu, Lin Li, Li-Wei Bai, Chang-Shui Xu

**Affiliations:** ^1^Department of Endocrinology, The First Affiliated Hospital of Xinxiang Medical University, Weihui, China; ^2^Department of Neurology, The First Affiliated Hospital of Xinxiang Medical University, Weihui, China; ^3^Department of Neurology, Henan Province People’s Hospital, Zhengzhou, China

**Keywords:** diabetic peripheral neuropathy, autophagy, oxidative stress, *XIST*, *miR-30d-5p*, SIRT1

## Abstract

Diabetic peripheral neuropathy (DPN) is a prevalent diabetes mellitus (Feldman et al., 2017) complication and the primary reason for amputation. Meanwhile, long non-coding RNAs (lncRNAs) are a type of regulatory non-coding RNAs (ncRNAs) that broadly participate in DPN development. However, the correlation of lncRNA X-inactive specific transcript (XIST) with DPN remains unclear. In this study, we were interested in the role of XIST in the modulation of DPN progression. Significantly, our data showed that the expression of *XIST* and sirtuin1 (SIRT1) was inhibited, and the expression of microRNA-30d-5p (*miR-30d-5p*) was enhanced in the trigeminal sensory neurons of the diabetic mice compared with the normal mice. The levels of LC3II and Beclin-1 were inhibited in the diabetic mice. The treatment of high glucose (HG) reduced the XIST expression in Schwann cells. The apoptosis of Schwann cells was enhanced in the HG-treated cells, but the overexpression of *XIST* could block the effect in the cells. Moreover, the levels of LC3II and Beclin-1 were reduced in the HG-treated Schwann cells, while the overexpression of *XIST* was able to reverse this effect. The HG treatment promoted the production of oxidative stress, while the *XIST* overexpression could attenuate this result in the Schwann cells. Mechanically, *XIST* was able to sponge *miR-30d-5p* and *miR-30d-5p*-targeted SIRT1 in the Schwann cells. *MiR-30d-5p* inhibited autophagy and promoted oxidative stress in the HG-treated Schwann cells, and SIRT1 presented a reversed effect. *MiR-30d-5p* mimic or SIRT1 depletion could reverse *XIST* overexpression-mediated apoptosis and autophagy of the Schwann cells. Thus, we concluded that *XIST* attenuated DPN by inducing autophagy through *miR-30d-5p*/SIRT1 axis. XIST and *miR-30d-5p* may be applied as the potential targets for DPN therapy.

## Introduction

Diabetic peripheral neuropathy (DPN) is a prevalent diabetes mellitus (Feldman et al., 2017) complication and is the principal reason for amputation (Feldman et al., 2017). Growing data have shown that Schwann cell dysfunction presents an essential role in DPN pathogenesis, such as slow migration speed, lipid metabolism abnormality, neurotrophy deficiency, and apoptosis (Naruse, 2019; Liu et al., 2020). However, the molecular mechanisms of DPN progression are poorly understood.

Autophagy is a changing process and controls cellular homeostasis by recovering miss-folded proteins and damaged organelles (Kuma et al., 2017). It is regarded as a protecting process that supports normal cell growth and function under physiological or pathological circumstances, particularly in metabolic diseases (Qu et al., 2016). During autophagy, organelles like endoplasmic reticulum and mitochondria are engulfed in a double-membrane-bound vesicle called autophagic vacuole or autophagosome, in which the outer membranes fuse with lysosome to produce autolysosomes, remodeling metabolic process ATP generation. Autophagy is critical for maintaining cellular homeostasis in adverse environments (Agrotis et al., 2019; Li and Zhang, 2019; Larabi et al., 2020). Autophagy was regulated by autophagy-related (Atg) factors. The core autophagy machinery comprises Atg12/Atg5 and Atg8 (LC3/GABARAP in mammals)E ubiquitin-like conjugation systems, Atg9 trafficking system, ULK protein kinase complex, and BECN1/beclin1-PIK3C3/Vps34 lipid kinase complex. The lipidation reaction of LC3 is catalyzed by E1-like activating enzyme Atg7 and E2-like conjugating enzyme Atg3 and is enhanced by the E3-like Atg12–Atg5 (Agrotis et al., 2019; Li and Zhang, 2019; Larabi et al., 2020). High glucose (HG) serves as the principal feature of DM, affecting the autophagy in different cells and tissues, including cardiomyocyte, podocyte, renal tubular cell, retina, and brain (Du et al., 2019). The autophagy inhibition has been identified in HG-treated Schwann cells and in diabetic models (Chung et al., 2018). However, the molecular mechanism of autophagy regulation in DPN is still unclear.

Different types of non-coding RNAs (ncRNAs) have been investigated in recent years, in which long non-coding RNAs (lncRNAs) serve as the transcripts of >200 nt and have no protein-coding ability (Mathy and Chen, 2017). LncRNAs present various biological functions, such as cell growth, cell differentiation, cell cycle, genomic imprinting, and dosage compensation, at the post-transcriptional, transcriptional, and epigenetic levels (Grote and Boon, 2018). Emerging investigations report that lncRNAs can mediate neural cell activities, regulating various CNS pathologies, including DPN (Andersen and Lim, 2018). Meanwhile, LncRNA X-inactive specific transcript (*XIST*) has been identified as an acknowledged cancer-associated regulator in several models (Zhao et al., 2020). Besides, it has been found that XIST is able to enhance autophagy processes (Xie et al., 2019). However, the effect of *XIST* on the autophagy of DPN is still obscure. MicroRNAs (miRNAs) are a type of endogenous, small, non-coding RNAs that regulate the target genes by specifically binding to the 3′ untranslated region (3′ UTR) at the post-transcriptional level (Saliminejad et al., 2019). Several studies have identified multiple miRNAs in the progression of DPN (Hu et al., 2019, 2020). Meanwhile, microRNA-30d-5p (miR-30d-5p) serves as a tumor suppressor and has the ability to induce cell apoptosis (Zeng et al., 2020). Moreover, Sirtuin1 (SIRT1) is a critical metabolic regulator and confers to the regulation of cellular NAD^+^/NADH ratio (Houtkooper et al., 2012). Importantly, it has been identified that SIRT1 is able to attenuate DPN progression (Chandrasekaran et al., 2019). However, whether *miR-30d-5p* and SIRT1 are involved in the modulation of *XIST*-mediated DPN remains elusive.

In this study, we explored the role of *XIST* in the regulation of the development of DPN. We elucidated an unreported function of *XIST* in attenuating DPN by modulating autophagy through *miR-30d-5p/*SIRT1 axis.

## Materials and Methods

### Cell Culture

The RSC96 cells were maintained in the lab and were incubated at 37°C with 5% CO_2_ in DMEM (General Electric, United States) containing fetal bovine serum (15%, Gibco, United States), streptomycin (0.1 mg/ml, Gibco, United States), and penicillin (100 units/ml, Gibco, United States). The lentiviral plasmids carrying *XIST* shRNA, SIRT1 shRNA, the control shRNA, the pcDNA3.1-*XIST* overexpression vector, pcDNA3.1-SIRT1 overexpression vector, *miR-30d-5p* mimic, and inhibitor were obtained (GenePharma, China; GenScript, China). The transfection in the cells was performed by Liposome 3000 (Invitrogen, United States). For the HG treatment, the RSC96 cells were cultured in DMEM with 150 mM D−glucose (Sigma, United States) for 48 h to simulate HG condition.

### Diabetic Peripheral Neuropathy Mouse Model

CD1 mice were obtained from the Chinese Academy of Medical Sciences (Beijing, China). Mice were randomly set into two groups, including normal group and diabetic group. The diabetic mice model was induced by intraperitoneally injecting streptozocin (150 mg/kg, Sigma, United States), and normal group mice were injected with sodium citrate solution. The serum glucose (16.7 mM) injected mice were regarded as diabetic mice. After 8 weeks, all mice were euthanized for further analysis. Animal care was authorized by the Animal Ethics Committee of the First Affiliated Hospital of Xinxiang Medical University. All experimental procedures with mice were performed in accordance and in compliance with the regulations of the Laboratory Animal Welfare and Ethics Committee.

### Analysis of Cell Apoptosis

About 2 × 10^6^ RSC96 cells were plated on 6-well dishes. Cell apoptosis was assessed by employing the Annexin V-FITC Apoptosis Detection Kit (Cell Signaling Technology, United States) according to the instruction of the manufacturer. Then, about 2 × 10^6^ collected and washed cells, collected using binding buffer, were dyed at 25°C, followed by flow cytometry analysis. The experiments were independently repeated three times.

### Luciferase Reporter Gene Assay

The luciferase reporter gene assays were carried out using the Dual-luciferase Reporter Assay System (Promega, United States). The cells were transfected with pmirGLO-XIST or pmirGLO-SIRT1, and miR-30d-5p mimic or control mimic using riboFECT^TM^ CP Transfection Kit (RiboBio, China), followed by the analysis of luciferase activities based on the Dual-luciferase Reporter Assay System (Promega, United States). As control, the luciferase activities of Renilla were measured. The experiments were independently repeated three times.

### Quantitative Reverse Transcription-PCR (qRT-PCR)

Total RNAs were extracted using TRIZOL (Invitrogen, United States). The first-strand cDNA was manufactured as per the instructions of the manufacturer by a two-step protocol (TaKaRa, China). The qRT-PCR was carried out by applying SYBR-Green (Takara, China). The efficiency curve was performed to validate the primer efficiency. The primer sequences are as follows: *XIST* forward: 5′-ACGCTGCATGTGTCCTTAG-3′, reverse: 5′-GAGCCTCTTATAAGCTGTTTG-3′; *miR-30d-5p* forward: 5′-GCCTGTAAACATCCCCGAC-3′; *SIRT1* forward: 5′- GTGCAGGTAGTTCCTCGGTG -3′, reverse: 5′-CACAAC TCACAGCATGCACAA-3′; *GAPDH* forward: 5′-AACGGATT TGGTCGTATTGGG-3′, reverse: 5′-CCTGGAAGATGGTGATG GGAT-3′; *U6* forward: 5′-CTCGCTTCGGCAGCACA-3′, reverse: 5′-AACGCTTCACGAATTTGCGT-3′.

### Western Blot Analysis

Total proteins were extracted from the cells using RIPA buffer (Cell Signaling Technology, United States) and quantified using the BCA Protein Quantification Kit (Abbkine, United States). The proteins at the same concentration were subjected to SDS-PAGE and were transferred to polyvinylidene fluoride (PVDF) membranes (Millipore, United States), followed by the incubation with 5% milk and with the primary antibodies at 4°C overnight. The corresponding secondary antibodies (Boster, China) were used for incubating the membranes at room temperature for 1 h, followed by the visualization using a chemiluminescence detection kit (Beyotime, China). The primary antibodies applied in this study comprise Bax (Abcam, United States), caspase3 (Abcam, United States), cleaved-caspase3 (Abcam, United States), caspase9 (Abcam, United States), cleaved-caspase9 (Abcam, United States), LC3I (Abcam, United States), LC3II (Abcam, United States), Beclin-1 (Abcam, United States), SIRT1 (Abcam, United States), and β-actin (Abcam, United States). The experiments were independently repeated three times.

### Reactive Oxygen Species Production Analysis

The cellular reactive oxygen species (ROS) production was analyzed using 7′-dichlorodihydrofluorescein diacetate (DCFH-DA) staining (Sigma-Aldrich, United States) according to the instruction of the manufacturer. Briefly, about 1 × 10^4^ cells were plated on 96-well black dishes in the standard culture medium and were cultured overnight. Cells were stained with DCFH-DA (100 μM), and the fluorescence intensity of different groups was analyzed by confocal analysis. The experiments were independently repeated three times.

### Statistical Analysis

Data were expressed as mean ± SD, and the statistical analysis was conducted using GraphPad prism 7. The unpaired Student’s *t*-test was used to compare two groups, and the one-way ANOVA was used to compare among multiple groups. *p* < 0.05 was considered statistically significant. The experiments were independently repeated three times.

## Results

### The Expression of *XIST* and SIRT1 and Autophagy Are Decreased, and the Expression of miR-30d-5p Is Increased in the Trigeminal Sensory Neurons of the Diabetic Mice

**FIGURE 1:**
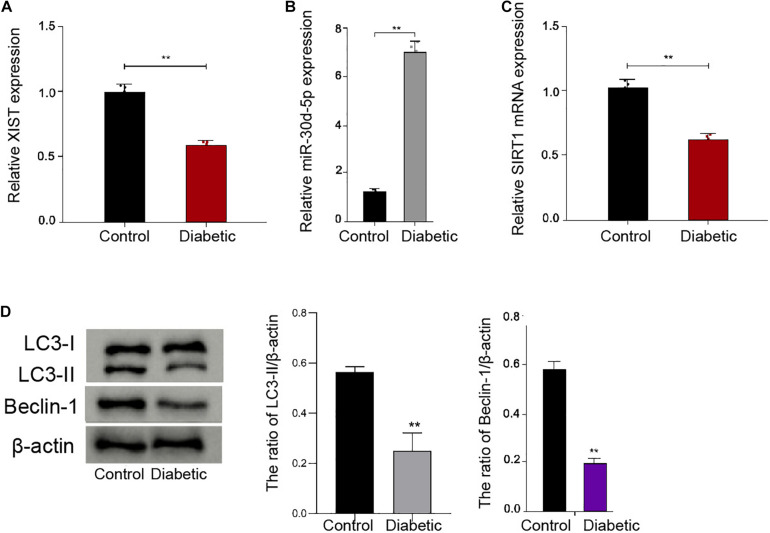
The expression of XIST and SIRT1 and autophagy are decreased, and the expression of miR-30d-5p is increased in the trigeminal sensory neurons of the diabetic mice. **(A)** The expression of XIST is measured by qPCR assays in the trigeminal sensory neurons of the diabetic mouse model. **(B)** The expression of miR-30d-5p is detected by qPCR assays in the trigeminal sensory neurons of the diabetic mouse model. **(C)** The expression of SIRT1 is examined by qPCR assays in the trigeminal sensory neurons of the diabetic mouse model. **(D)** The expression of LC3I, LC3II, and Beclin-1 is assessed by Western blot analysis in the trigeminal sensory neurons of the diabetic mouse model. *N* = 6, mean ± SD, **P < 0.01.

To analyze the potential correlation of *XIST*, *miR-30d-5p*, and SIRT1 with DPN *in vivo*, we established a diabetic mouse model and assessed its expression in the trigeminal sensory neurons of the mice. We observed that the expression of *XIST* and SIRT1 was inhibited, while the miR-30d-5p expression was induced in the trigeminal sensory neurons of the diabetic mice compared with the normal mice (Figures 1A–C). Significantly, the ratio of LC3II/LC3I and the levels of Beclin-1 were reduced in the trigeminal sensory neurons of the diabetic mice compared with the normal mice (Figure 1D), implying that autophagy was decreased in the trigeminal sensory neurons of the diabetic mice.

### *XIST* Attenuates High Glucose-Induced Apoptosis in the Schwann Cells

**FIGURE 2:**
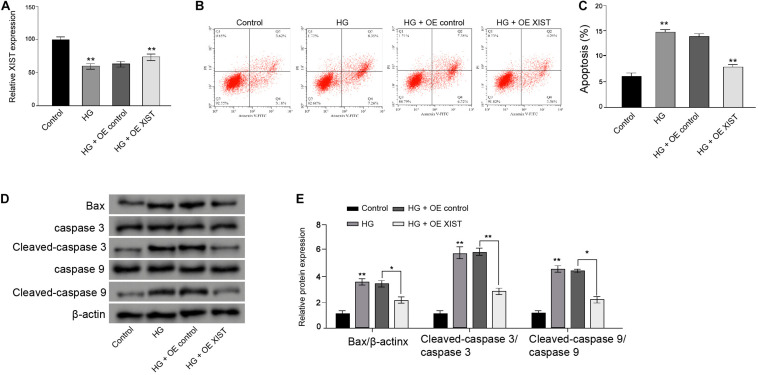
XIST attenuates high glucose-induced apoptosis in the Schwann cells. **(A–D)** The RSC96 cells are treated with HG, or co-treated with HG and pcDNA-3.1-XIST overexpression vectors. **(A)** The expression of XIST is measured by qPCR assays. **(B,C)** The apoptosis is detected by flow cytometry analysis. **(D,E)** The expression of Bax, caspase3, cleaved-caspase3, caspase9, and cleaved-caspase9 is assessed by Western blot analysis. *N* = 3, mean ± SD, *P < 0.05, **P < 0.01.

Next, the RSC96 rat Schwann cells underwent a HG treatment and were treated with *XIST* overexpression vectors. The HG treatment reduced the *XIST* expression, while the *XIST* overexpression vectors enhanced the *XIST* levels in the RSC96 cells (Figure 2A). Moreover, apoptosis of RSC96 cells was induced in the HG-treated cells, but the overexpression of *XIST* could block the effect in the cells (Figures 2B,C). Meanwhile, the expression of Bax, cleaved-caspase3, and cleaved-caspase9 was enhanced by HG, in which the *XIST* overexpression reverses this phenotype in the RSC96 cells (Figures 2D,E).

### *XIST* Induces Autophagy and Inhibits Oxidative Stress in the HG-Treated Schwann Cells

**FIGURE 3:**
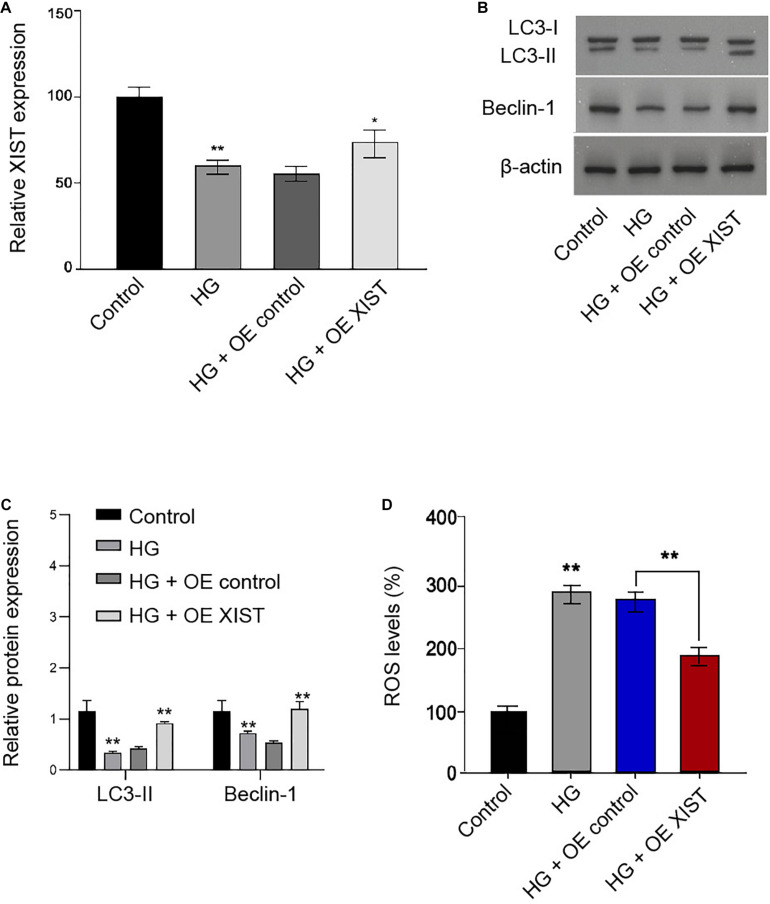
XIST induces autophagy and inhibits oxidative stress in the HG-treated Schwann cells. **(A–D)** The RSC96 cells are treated with HG, or co-treated with HG and pcDNA-3.1-XIST overexpression vectors. **(A)** The expression of XIST is measured by qPCR assays. **(B,C)** The expression of LC3I, LC3II, and Beclin-1 is assessed by Western blot analysis. **(D)** DCFH–DA staining shows the ROS production. *N* = 3, mean ± SD, *P < 0.05, **P < 0.01.

Next, we validated that *XIST* overexpression vectors rescued HG treatment-inhibited *XIST* expression in the RSC96 cells (Figure 3A). Moreover, the ratio of LC3II/LC3I and the levels of Beclin-1 were reduced in the HG-treated RSC96 cells, in which the overexpression of *XIST* was able to reverse this effect in the cells (Figures 3B,C). In addition, the HG treatment induced the production of ROS, while the *XIST* overexpression could attenuate this result in the RSC96 cells (Figure 3D).

### *XIST* Is Able to Sponge *miR-30d-5p* in the Schwann Cells

**FIGURE 4:**
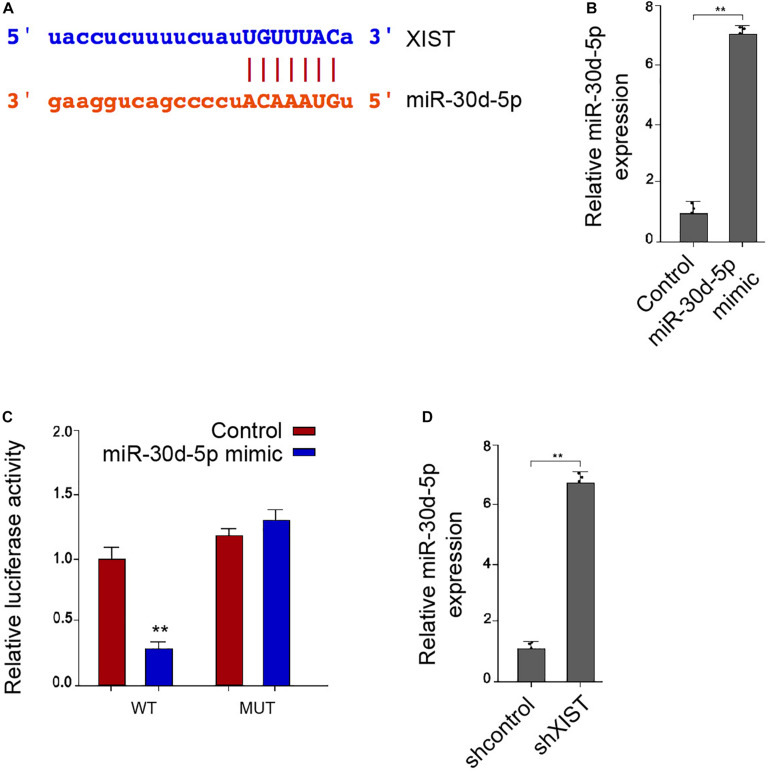
XIST is able to sponge *miR-30d-5p* in the Schwann cells. **(A)** Bioinformatic analysis using ENCORI (http://starbase.sysu.edu.cn/index.php) shows the interaction of XIST with miR-30d-5p. **(B,C)** The RSC96 cells are treated with control mimic or miR-30d-5p mimic. **(B)** The expression of miR-30d-5p is detected by qPCR assays. **(C)** The luciferase activities of the wild-type XIST (WT) or mutant XIST (MUT) are detected by luciferase reporter gene assays. **(D)** The RSC96 cells are treated with control shRNA or XIST shRNA. The expression of miR-30d-5p is detected by qPCR assays. *N* = 3, mean ± SD, **P < 0.01.

Interestingly, bioinformatic analysis showed a potential correlation of *XIST* with *miR-30d-5p* (Figure 4A). We confirmed that *miR-30d-5p* mimic was able to enhance the *miR-30d-5p* expression in the RSC96 cells (Figure 4B). Remarkably, the *miR-30d-5p* mimic reduced the luciferase activities of the wild-type *XIST* in the RSC96 cells (Figure 4C). As expected, the expression of *miR-30d-5p* was up-regulated by *XIST* depletion in the cells (Figure 4D).

### *MiR-30d-5p* Inhibits Autophagy and Promotes Oxidative Stress in the HG-Treated Schwann Cells

**FIGURE 5:**
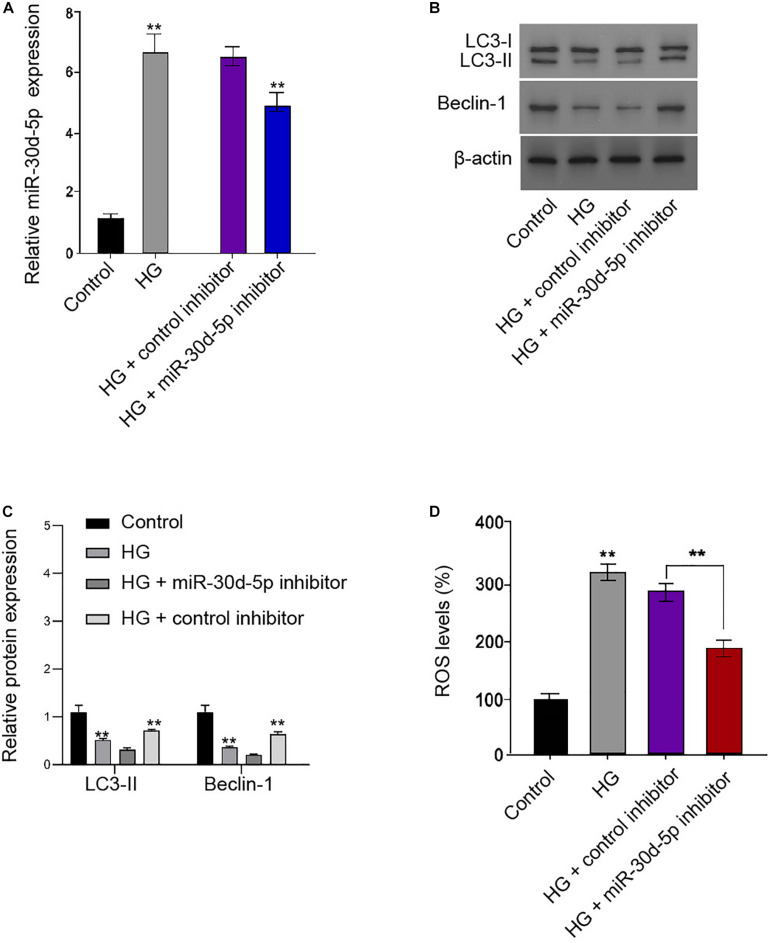
MiR-30d-5p inhibits autophagy and promotes oxidative stress in the HG-treated Schwann cells. **(A–D)** The RSC96 cells are treated with HG, or co-treated with HG and miR-30d-5p inhibitor. **(A)** The expression of miR-30d-5p is detected by qPCR assays. **(B,C)** The expression of LC3I, LC3II, and Beclin-1 is assessed by Western blot analysis. **(D)** DCFH–DA staining shows the ROS production. *N* = 3, mean ± SD, *P < 0.05, **P < 0.01.

We further showed that *miR-30d-5p* was enhanced in the HG-treated RSC96 cells, and the efficiency of miR-30d-5p inhibitor was verified in the cells (Figure 5A). Functionally, the ratio of LC3II/LC3I and the levels of Beclin-1 were inhibited in the HG-treated RSC96 cells, in which *miR-30d-5p* inhibitor reversed this impact in the cells (Figures 5B,C). Besides, the HG treatment enhanced the production of ROS, and miR-30d-5p inhibitor could alleviate this phenotype in the RSC96 cells (Figure 5D).

### *MiR-30d-5p* Is Able to Target SIRT1 in the Schwann Cells

**FIGURE 6:**
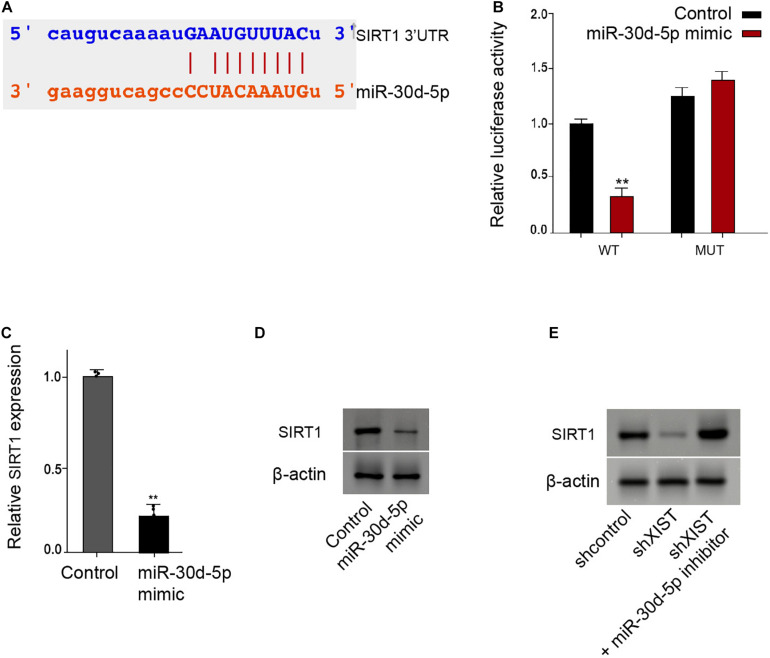
MiR-30d-5p is able to target SIRT1 in the Schwann cells. **(A)** Bioinformatic analysis using Targetscan (http://www.targetscan.org/vert_72/) shows the interaction of SIRT1 with miR-30d-5p. **(B–D)** The RSC96 cells are treated with control mimic or miR-30d-5p mimic. **(B)** The luciferase activities of wild-type SIRT1 (WT) or mutant SIRT1 (MUT) are detected by luciferase reporter gene assays. **(C)** The expression of SIRT1 is examined by qPCR assays. **(D)** The expression of SIRT1 is examined by Western blot analysis. **(E)** The RSC96 cells are treated with control shRNA or XIST shRNA, or co-treated with XIST shRNA and miR-30d-5p inhibitor. The expression of SIRT1 is examined by Western blot analysis. *N* = 3, mean ± SD, *P < 0.05, **P < 0.01.

Moreover, we identified the binding site of SIRT1 3′ UTR with the *miR-30d-5p* (Figure 6A). The *miR-30d-5p* mimic reduced the luciferase activities of the wild-type SIRT1 in the RSC96 cells (Figure 6B). Consistently, the mRNA and protein levels of SIRT1 were down-regulated by the *miR-30d-5p* mimic in the RSC96 cells (Figures 6C,D). Meanwhile, the depletion of *XIST* reduced the SIRT1 expression, in which *miR-30d-5p* inhibitor could reverse this effect in the cells (Figure 6E).

### SIRT1 Induces Autophagy and Inhibits Oxidative Stress in the HG-Treated Schwann Cells

**FIGURE 7:**
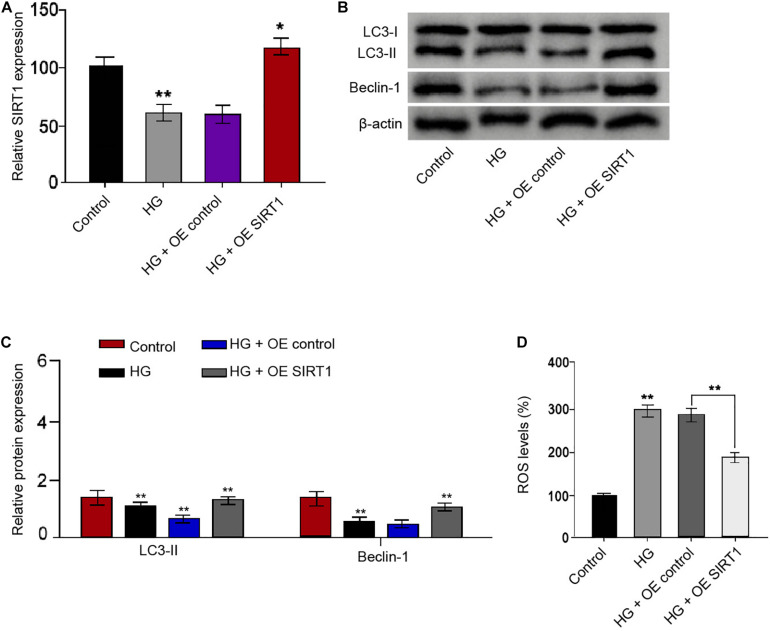
SIRT1 induces autophagy and inhibits oxidative stress in the HG-treated Schwann cells. **(A–D)** The RSC96 cells are treated with HG, or co-treated with HG and pcDNA-3.1-SIRT1 overexpression vectors. **(A)** The expression of SIRT1 is examined by qPCR assays. **(B,C)** The expression of LC3I, LC3II, and Beclin-1 is assessed by Western blot analysis. **(D)** DCFH–DA staining shows the ROS production. *N* = 3, mean ± SD, *P < 0.05, **P < 0.01.

We then identified that SIRT1 overexpression vectors rescued the HG treatment-attenuated SIRT1 expression in the RSC96 cells (Figure 7A). The ratio of LC3II/LC3I and the levels of Beclin-1 were reduced in the HG-treated RSC96 cells, while the overexpression of SIRT1 was able to reverse this effect in the cells (Figures 7B,C). Moreover, the HG treatment induced the production of ROS, while the SIRT1 overexpression could reverse this induction in the RSC96 cells (Figure 7D).

### *XIST* Attenuates HG-Induced Apoptosis and Induces Autophagy by *miR-30d-5p*/SIRT1 Axis in the HG-Treated Schwann Cells

**FIGURE 8:**
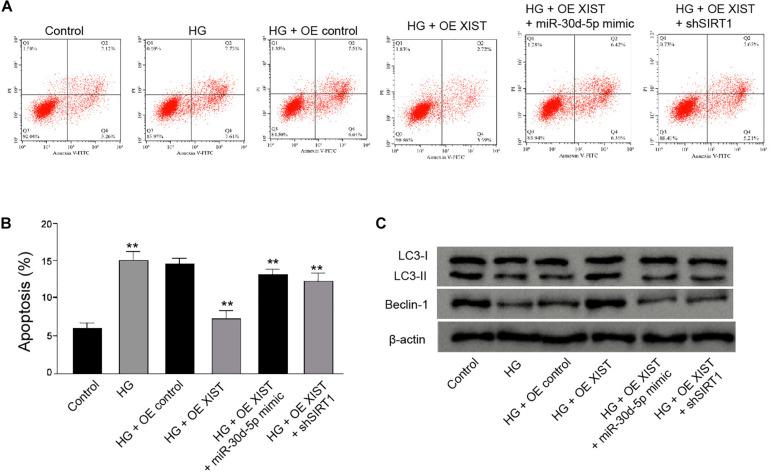
XIST attenuates high glucose-induced apoptosis and induces autophagy by miR-30d-5p/SIRT1 axis in the HG-treated Schwann cells. **(A–C)** The HG-treated RSC96 cells are treated with pcDNA-3.1-XIST overexpression vectors, or co-treated with pcDNA-3.1-XIST overexpression vectors and miR-30d-5p mimic or SIRT1 shRNA. **(A,B)** The apoptosis is detected by flow cytometry analysis. **(C)** The expression of LC3I, LC3II, and Beclin-1 is assessed by Western blot analysis. *N* = 3, mean ± SD, **P < 0.01.

Furthermore, we found that the *XIST* overexpression attenuated HG-induced apoptosis of RSC96 cells, while the *miR-30d-5p* mimic or SIRT1 depletion could rescue this phenotype in the cells (Figures 8A,B). Meanwhile, the *XIST* overexpression enhanced the ratio of LC3II/LC3I and the levels of Beclin-1 in the HG-treated RSC96 cells, in which *miR-30d-5p* mimic or SIRT1 depletion was able to reverse this effect (Figure 8C).

## Discussion

Diabetic peripheral neuropathy (DPN) is the prevailing complication of DM and contributes to the occurrence of amputation, in which autophagy dysfunction plays crucial roles. LncRNAs have been found to exert important functions in the regulation of DPN. Nevertheless, the effect of *XIST* on the progression of DPN is still unreported. In this study, we first identified that *XIST* attenuated DPN by inducing autophagy through *miR-30d-5p*/SIRT1 axis.

Previous studies have found several lncRNAs in the DPN regulation. It has been reported that PVT1 protects DPN via PI3K/AKT pathway (Chen et al., 2018). LncRNA HCG18 enhances the M1 polarization of the macrophages by regulating the miR-146a/TRAF6 signaling, contributing to DPN progression (Ren et al., 2020). LncRNA uc.48 + participates in DPN regulated by the P2 × 3 receptor (Wang et al., 2016). LncRNAs modulate inflammation in DPN by targeting miR-146a-5p (Feng et al., 2020). Meanwhile, it has been well-identified that the induction of autophagy is able to alleviate DPN (Chung et al., 2018; Dewanjee et al., 2018). Meanwhile, it has been reported that the phosphorylation of STAT3 regulates HG-attenuated autophagy in DPN by regulating HDAC1, and HG affects the autophagy in podocytes (Du et al., 2019). Besides, *XIST* regulates HG-induced podocyte injury by targeting miR-30/AVEN in diabetic nephropathy (Long et al., 2020). In this study, we found that *XIST* expression was inhibited in the trigeminal sensory neurons of the diabetic mice compared with the normal mice. Autophagy was decreased in the trigeminal sensory neurons of the diabetic mice. *XIST* attenuated HG-induced apoptosis in the Schwann cells. *XIST* induced autophagy and inhibited oxidative stress in the HG-treated Schwann cells. Our finding identifies an important role of *XIST* in regulating DPN, presenting valuable information on the crucial functions of lncRNAs in DPN progression. These results provide a new evidence of lncRNA *XIST* in regulating DPN, enriching the understanding of the molecular mechanisms of the roles of lncRNA in DPN. Meanwhile, it improves the knowledge about the function of lncRNA-mediated autophagy in DPN progression, emphasizing the critical effect of autophagy on DPN.

Moreover, miRNAs are widely involved in the regulation of DPN. It has been reported that miR-34c regulates DPN by modulating autophagy (Hu et al., 2019). The suppression of miR-25 enhances DPN (Zhang et al., 2018). MiR-146a modulates DPN by targeting thymosin β4-induced neurovascular remodeling in mice (Wang et al., 2019). MiR-146a mediates DPN by the modulation of inflammation (Feng et al., 2018). Moreover, it has been found that SIRT1 is able to attenuate DPN in several previous reports (Yerra et al., 2017; Chandrasekaran et al., 2019; Zhang et al., 2019). Our data showed that *miR-30d-5p* inhibited autophagy and promoted oxidative stress in the HG-treated Schwann cells. SIRT1 induced autophagy and inhibited oxidative stress in the HG-treated Schwann cells. XIST was able to enhance SIRT1 expression by targeting miR-30d-5p. And miR-30d-5p mimic or SIRT1 depletion could reverse XIST overexpression-mediated apoptosis and autophagy of Schwann cells. These data elucidate an unreported association of *XIST* with SIRT1 and *miR-30d-5p*, presenting a new mechanism involving *XIST*, *miR-30d-5p*, and SIRT1 in DPN pathogenesis. Meanwhile, it validates the crucial function of SIRT1 in regulating autophagy during DPN, which is consistent with the previous report (Yerra et al., 2017). Our finding also provides a new evidence that miRNAs are involved in the regulation of autophagy in DPN. Moreover, *miR-30d-5p/*SIRT1 axis may just be one of the downstream mechanisms of *XIST-*mediated DPN, and more potentially, mechanisms and their correlation with *XIST/miR-30d-5p/*SIRT1 axis in DPN are needed to explore in the future.

## Conclusion

In conclusion, we discovered that *XIST* attenuated DPN by inducing autophagy through *miR-30d-5p*/SIRT1 axis. *XIST* and *miR-30d-5p* may be applied as the potential targets for DPN therapy.

## Data Availability Statement

The original contributions presented in the study are included in the article/supplementary material, further inquiries can be directed to the corresponding author/s.

## Ethics Statement

The animal study was reviewed and approved by the First Affiliated Hospital of Xinxiang Medical University.

## Author Contributions

B-YL designed the study. L-WB performed the experiments. LL collected and analyzed the data. C-SX wrote the manuscript. All authors contributed to the article and approved the submitted version.

## Conflict of Interest

The authors declare that the research was conducted in the absence of any commercial or financial relationships that could be construed as a potential conflict of interest.
